# Incomplete Medical Charts: Impacts And Possible Solutions

**DOI:** 10.1192/j.eurpsy.2022.1930

**Published:** 2022-09-01

**Authors:** M. Chowdhury, S. Peteru, A. Askandaryan, D. Banik, J. Hiana

**Affiliations:** 1 JAMAICA HOSPITAL MEDICAL CENTER, Psychiatry, NEW YORK, United States of America; 2 ST. JOHN’S EPISCOPAL HOSPITAL, Psychiatry, FAR ROCKAWAY, United States of America; 3 SUNY DOWNSTATE MEDICAL CENTER, Neurology, BROOKLYN, United States of America

**Keywords:** Solutions, Incomplete Charts, Impacts

## Abstract

**Introduction:**

Proper documentation and relevant updating of patients’ health status has become a cumbersome task with the inception of electronic medical records.Inpatient, ED, and ambulatory patient evaluation generate billions of records each year.It brings about a burden on the workload of the providers regarding registering and completing patients’ records.Incomplete medical records set up complications in patient management and subsequent administrative operations.Specifically, denials for reimbursement because of incomplete medical records emerge as a critical concern.Effective measures, consisting of both technical and administrative enforcements are required to reduce number of open charts.

**Objectives:**

To understand the reasons,consequences and solutions for Incomplete/Delinquent medical records.

**Methods:**

We searched Google scholar and Pubmed database using keywords “Incomplete medical records”, “Imapacts” and “Solutions”.Articles popped up.We selected 4 based on internal and external validity.

**Results:**

Incomplete/Delinquent medical records are nowadays imposing a critical challenge upon financial, administrative and legal affairs in practicing Medicine.Our review shows that CMS recovery audit with hospital denials went high from 7-10% in recent years because of open/incomplete medical records.Provider’s documentation time increases as well with implementation of EHR.Appropriate measures could be taken to resolve this issue, broadly we can try two ways- training and administrative courses.

**Conclusions:**

Physicians,residents and all respective providers should get training on a regular basis regarding EMR/EHR to complete medical records duly and effectively.The other way is administrative surveillance.If providers fail repeatedly to comply with HIM standard and complete delinquent medical records, warnings, suspensions or other regulatory actions can be executed strictly to keep them on track.

**Disclosure:**

No significant relationships.

